
JOURNAL INFORMATION
==============================
NLM Title Abbreviation: Behav Neurol
Journal ID: Behav Neurol
NLM Journal ID: 8914585
Journal ID: BN

Behavioural Neurology

ISSN: 0953-4180
EISSN: 1875-8584
Publisher: Wiley

ARTICLE INFORMATION
==============================
PMCID: PMC5213552
PMID: 22713402
DOI: 10.3233/BEN-2012-120259
Article ID: 728616
Article version: 1
Subjects: Introduction

Deterioration or Recovery of Selective Cognitive Function Can Reveal the Role of Focal Areas within Networks of the Brain

Hillis Argye E. 1 2 3 *
1 Departments of Neurology Johns Hopkins University School of Medicine Baltimore MD USA
2 Physical Medicine and Rehabilitation Johns Hopkins University School of Medicine Baltimore MD USA
3 Department of Cognitive Science Johns Hopkins University Baltimore MD USA
*Argye E. Hillis: argye@jhm1.edu
Print publication date: 2013
Volume: 26
Issue: 1-2
First page: 3
Last page: 5
Received 2012 Apr 23; Accepted 2012 Apr 23
Copyright: Copyright © 2013 Hindawi Publishing Corporation and the authors.
Copyright year: 2013
License: This is an open access article distributed under the Creative Commons Attribution License, which permits unrestricted use, distribution, and reproduction in any medium, provided the original work is properly cited.
License URL: https://creativecommons.org/licenses/by/3.0/

Funding: http://dx.doi.org/10.13039/100000055 National Institute on Deafness and Other Communication Disorders R01 DC05375 http://dx.doi.org/10.13039/100000055 National Institute on Deafness and Other Communication Disorders R01 DC 03681

==============================
Behavioural Neurology 26 (2013) 3–5
DOI 10.3233/BEN-2012-120259
IOS Press

Introduction

### Deterioration or recovery of selective cognitive function can reveal the role of focal areas within networks of the brain

Argye E. Hillis[a,b,c,*]
[a]*Departments of Neurology, Johns Hopkins University School of Medicine, Baltimore, MD, USA*
[b]*Physical Medicine and Rehabilitation, Johns Hopkins University School of Medicine, Baltimore, MD, USA*
[c]*Department of Cognitive Science, Johns Hopkins University, Baltimore, MD, USA*

This double special issue of Behavioural Neurology is devoted to two topics that have traditionally captured the most interest of behavioural neurologists – aphasia and hemispatial neglect. In recent years, more time and literature of behavioural neurologists has been devoted to more generalized dementia such as Alzheimer's Disease, perhaps because that is "where the money is" or because aphasia and neglect are typically associated with stroke, and stroke neurology has become a somewhat independent field of neurology (or medicine) focused on recanalization of vessels rather than behaviour or localization of deficits. Some of us have investigated how restoration of blood flow in acute stroke in particular areas of brain affects aphasia or neglect [1–3, 5], but there has not been a great deal of overlap in the acute stroke and behavioural neurology literature in the last few decades [but see 6–9 for notable examples of overlap].

The special issue on aphasia includes a number of papers that compare post-stroke aphasia with Primary Progressive Aphasia (PPA), a relatively recently described focal degeneration syndrome in which the predominant deficit is language (with or without apraxia) for the first two years [15]. Three variants have been described that are fairly regularly associated with distinct underlying pathologies, as well as distinct areas of brain atrophy [16]. The nonfluent, agrammatic variant of PPA (nfvPPA, also called PPA-G emphasising that grammar is frequently impaired, or formerly called progressive nonfluent aphasia, emphasing that speech articulation is often slow, effortful and distorted). The nonfluent/agrammatic variant is associated with atrophy in the posterior inferior frontal cortex and insula and most often with the tau pathology, such as corticobasal degeneration, progressive supranuclear palsy, or frontotemporal lobar degeneration-tau at autopsy [see 16 for review]. The key features are agrammatic language production and/or apraxia of speech. Verb naming is typically more impaired than noun naming [4]. Thus, it has a great deal of overlap with the vascular syndrome of Broca's aphasia (not surprisingly, as overlapping areas of the brain are affected). Unlike the vascular syndrome, there are some patients with nfvPPA who have spared writing until late in the course [see 2]. The semantic variant of PPA (svPPA or PPA-S, also sometimes called semantic dementia) is associated with atrophy in the anterior and inferior temporal cortex (left greater than right) and is generally found to be associated with ubiquitin pathology at autopsy. The key feature is impaired word comprehension with or without impaired object recognition but relatively spared motor speech, grammar and fluency of language production, and sentence repetition. Individuals with svPPA show overlap in language performance with in-

*Correspondence to: Argye E. Hillis, MD, Meyer 6-113, Johns Hopkins Hospital, Baltimore, MD 21287, USA. Tel.: +1 410 614 2381; Fax: +1 410 955 0672; E-mail: argye@JHMI.edu.

ISSN 0953-4180/13/$27.50 © 2013 – IOS Press and the authors. All rights reserved dividuals with the vascular syndrome of Wernicke's aphasia. Again, this is not surprising, as both syndromes are associated primarily with left temporal lobe damage or dysfunction. However, unlike individuals with post-stroke aphasia, most individuals with svPPA do show object agnosia at least late in the course or at least with lower familiarity objects, perhaps because they have some bilateral temporal cortex involvement. Finally, logopenic variant PPA (lvPPA or PPA-L) is associated with posterior superior temporal and inferior parietal cortex atrophy (left much greater than right) and Alzheimer's disease (AD) pathology at autopsy. It is characterised by impaired naming with phonological errors and disproportionately impaired sentence repetition (e.g. compared to sentence reading). Reduced phonological working memory is the underlying cognitive process that is impaired in most cases. This variant of PPA has a great deal of overlap with the vascular syndrome of conduction aphasia, at least at onset. Eventually, individuals often develop more severe language or memory deficits consistent with AD.

Comparing post-stroke aphasia and PPA is a useful enterprise for a number of reasons. First, investigation of vascular syndromes alone is limited by the human vasculature and its territories. That is, not all areas of the brain are equally likely to be affected by stroke, and some areas are rarely involved in stroke because they have redundant vascular supplies. PPA frequently affects some of these areas, allowing investigator to test the role of these areas in language. Likewise, stroke is generally unilateral, while PPA is typically asymmetric, but bilateral (so the role of each hemisphere can be better tested in the presence of damage to one vs both hemispheres). Deficits from stroke often recover, while deficits in PPA progress over time. The course of breakdown or recovery often reveals interesting insights about the cognitive mechanisms underlying the affected language tasks.

In this issue Lambon-Ralph and co-authors report results of an investigation of nonverbal semantic processing in svPPA and post-stroke aphasia with semantic deficits. Faria and collegues compare patterns of dysgraphia (spelling impairment) in PPA, and compare them to patterns of dysgraphia previously described in individuals with post-stroke aphasia. They also report the areas of focal atrophy associated with the most common pattern of dysgraphia in PPA. In another paper on dysgraphia in PPA versus stroke, Tsapkini and colleagues report treatment of spelling in one individual with lvPPA, and compare treatment results to the same approach to treatment of spelling in an individual with post-stroke dysgraphia. Meltzer-Asscher et al. evaluate the distinct patterns of morphosyntactic errors in the variants of PPA, and compare them to patterns of errors in post-stroke aphasia. Weintraub and co-workers report results of a new method of assessment of verbal and nonverbal memory in PPA. The issue is completed by a three Clinical Notes: a fascinating case of an unusual form of lvPPA that degenerated into jargon aphasia, a case of nonfluent agrammatic variant PPA due to Pick disease with concomitant (what is argued to be) incidental Alzheimer's disease pathology, and a case of successful treatment of PPA. Together, these papers illustrate how investigating PPA and post-stroke aphasia can yield complementary insights about brain-behaviour relationships as well as about potential response to interventions and the normal cognitive processes underlying language. The separate introduction to the special issue on hemispatial neglect and related disorders describes the papers therein and how they contribute additional insights into brain-behaviour relationships and the cognitive processes underlying normal spatial attention and representation.

### Acknowledgments

The author and the some of the work reported herein were supported by NIH/NIDCD R01 DC05375 and R01 DC 03681.

### References

[1] A. Croquelois, M. Wintermark, M. Reichhart, R. Meuli and J. Bogousslavsky, Aphasia in hyperacute stroke: language follows brain penumbra dynamics, *Ann Neurol* **54** (2003), 321–329.

[2] A.E. Hillis, A. Kane, E. Tuffiash, J.A. Ulatowski, P. Barker, N. Beauchamp and R. Wityk, Reperfusion of specific brain regions by raising blood pressure restores selective language functions in subacute stroke, *Brain Lang* **79** (2002), 495–510.

[3] A.E. Hillis, K.T. Kleinman, M. Newhart, J. Heidler-Gary, R. Gottesman, P.B. Barker, E. Aldrich, R. Llinas, R. Wityk and P. Chaudhry, Restoring cerebral blood flow reveals neural regions critical for naming, *J Neurosci* **26** (2006), 8069–8073.

[4] A.E. Hillis, S. Oh and L. Ken, Deterioration of naming nouns versus verbs in primary progressive aphasia, *Ann Neurol* **55** (2004), 268–275.

[5] S. Khurshid, L.A. Trupe, M. Newhart, C. Davis, J. Molitoris, J. Medina, R. Leigh and A.E. Hillis, Reperfusion of specific cortical areas is associated with improvement in distinct forms of hemispatial neglect, Cortex. 2011 [Epub ahead of print].

[6] M. Lee, J.L. Saver, J.R. Alger, Q. Hao, N. Salamon, S. Starkman, L.K. Ali, B. Ovbiagele, D. Kim, J.P. Villablanca, M.T. Froehler, M.S. Tenser and D.S. Liebeskind, Association of laterality and size of perfusion lesions on neurological deficit in acute supratentorial stroke. Int J Stroke 2011; [Epub ahead of print].

[7] J.M. Ringman, J.L. Saver, R.F. Woolson, W.R. Clarke and H.P. Adams, Frequency, risk factors, anatomy, and course of unilateral neglect in an acute stroke cohort, *Neurology* **63**(3) (2004), 468–474.

[8] D. Saur, R. Lange, A. Baumgaertner, V. Schraknepper, K. Willmes, M. Rijntjes and C. Weiller, Dynamics of language reorganization after stroke, *Brain* **129** (2006), 1371–1384.

[9] D. Saur, O. Ronneberger, D. Kummerer, I. Mader, C. Weiller and S. Kloppel, Early functional magnetic resonance imaging activations predict language outcome after stroke, *Brain* **133** (2010), 1252–1264.

[10] A. Faria, J. Crinion, S. Cooley, K. Tsapkini, L.A. Trupe, S. Mori and A.E. Hillis, Patterns of Dysgraphia in Primary Progressive Aphasia Compared to Post-Stroke Aphasia, *Behavioural Neurology* **26** (2013), 21–34.

[11] L. Ralph, Demonstrating the qualitative differences between semantic aphasia and semantic dementia: A novel exploration of nonverbal semantic processing Meltzer-Asscher, Syntactic and morphosyntactic processing in stroke-induced and primary progressive aphasia, *Behavioural Neurology* **26** (2013), 7–20.

[12] P. Caffarra, S. Gardini, S. Cappa, F. Dieci, L. Concari, C. Caterina Ghetti, L. Ruffini and G. Dalla Rosa Prati, Degenerative jargon aphasia: unusual progression of logopenic/phonological progressive aphasia? *Behavioural Neurology* **26** (2013), 89–93.

[13] K. Tsapkini and A.E. Hillis, Spelling intervention in post-stroke aphasia and Primary Progressive Aphasia, *Behavioural Neurology* **26** (2013), 55–66.

[14] S. Weintraub, E. Rogalski, E. Shaw, S. Salwani, A. Rademaker, C. Wieneke and M. Marsel Mesulam, Verbal and nonverbal memory in primary progressive aphasia: the three words three shapes test, *Behavioural Neurology* **26** (2013), 67–76.

[15] M.M. Mesulam, Slowly progressive aphasia without generalized dementia, *Ann Neurol* **11**(6) (1982), 592–598.

[16] M.L. Gorno-Tempin, A.E. Hillis, S. Weintraub, A. Kertesz, M. Mendez, S. Cappa, J. Ogar, J. Rohrer, S. Black, B. Boeve, F. Manes, N. Dronkers, R. Vandenberghe, K. Rascovsky, K. Patterson, B. Miller, D. Knopman, J.R. Hodges, M. Mesulam and M. Grossman, Classification of primary progressive aphasia and its variants, *Neurology* **76**(11) (2011), 1006–1014.
